# Pathogen genomic surveillance of typhoidal *Salmonella* infection in adults and children reveals no association between clinical outcomes and infecting genotypes

**DOI:** 10.1186/s41182-020-00247-2

**Published:** 2020-07-13

**Authors:** Carl D. Britto, Sitarah Mathias, Ashish Bosco, Zoe A. Dyson, Gordon Dougan, Savitha Raveendran, V. L. Abin, Sanju Jose, Savitha Nagaraj, Kathryn E. Holt, Andrew J. Pollard

**Affiliations:** 1 Oxford Vaccine Group, Department of Paediatrics, University of Oxford and the NIHR Oxford Biomedical Research Centre, Oxford, OX3 7LE UK; 2 St. John’s Medical College Hospital and Division of Infectious Disease, St. John’s Research Institute, Bangalore, India; 3 Department of Infectious Diseases, Central Clinical School, Monash University, Melbourne, Victoria 3004 Australia; 4 Department of Medicine, University of Cambridge, Cambridge, UK; 5 Wellcome Trust Sanger Institute, Wellcome Genome Campus, Hinxton, UK; 6 London School of Hygiene & Tropical Medicine, London, WC1E 7HT UK; 7 Division of Infectious Disease, St. John’s Research Institute, Bengaluru, 560034 India

**Keywords:** Enteric fever, Typhi, Paratyphi, Clinical features, H58, India

## Abstract

**Background:**

India is endemic for enteric fever, and it is not known whether the variations in clinical manifestations between patients are due to host, environmental or pathogen factors.

Blood culture surveillance was conducted at St. John’s Medical College Hospital, Bangalore, between July 2016 and June 2017. Clinical, laboratory and demographic data were collected from each case, and bacterial isolates were subjected to whole genome sequencing. Comparative analysis between adults and paediatric patients was carried out to ascertain differences between adult and paediatric disease.

**Results:**

Among the 113 cases of blood culture-confirmed enteric fever, young adults (16–30 years) and children < 15 years accounted for 47% and 37% of cases, respectively. Anaemia on presentation was seen in 46% of cases, and 19% had an abnormal leucocyte count on presentation. The majority received treatment as inpatients (70%), and among these, adults had a significantly longer duration of admission when compared with children (*p* = 0.002). There were atypical presentations including arthritis, acute haemolysis and a case of repeated typhoid infection with two separate *S.* Typhi genotypes. There was no association between infecting genotype/serovar and treatment status (outpatient vs inpatient), month of isolation, duration of admission, patient age (adult or child), antimicrobial susceptibility, Widal positivity or haematologic parameters.

**Conclusions:**

Amidst the many public health concerns of South India, enteric fever continues to contribute substantially to hospital burden with non-specific as well as uncommon clinical features in both paediatric and adult populations likely driven by host and environmental factors. Robust clinical surveillance as well monitoring of pathogen population structure is required to inform treatment and preventive strategies.

## Background

The Indian subcontinent is endemic for enteric fever, a febrile illness caused by *Salmonella enterica* serovars Typhi (*S.* Typhi) and Paratyphi A (*S.* Paratyphi A), and on a global scale causes an estimated 14 million cases of febrile illness annually [1], disproportionally affecting children and young adults [2–4].

The most common circulating genotype of *S.* Typhi in South Asia is H58 (4.3.1) [5]. Longitudinal as well as a cross-sectional data from two separate hospital-based studies in Southern India confirmed that 4.3.1 was the dominant genotype in both settings, making up 77.8% and 88.2 % of the pathogen population in the respective study sites [6, 7].

Here, we present clinical data from one of these studies (Bangalore, 2016–2017), including the age distribution and clinical phenotype of patients confirmed with enteric fever. We highlight rare presentations, contrasting features of adult and paediatric enteric fever, and investigate the association of these with the molecular structure of the pathogen population.

## Main text

### Methods

This hospital-based surveillance took place in a tertiary care setting in Bangalore, South India, as described previously [7]. For the purpose of this study, between June 2016 and June 2017, every microbiological specimen (113 blood cultures and 1 joint aspirate) that was positive for a typhoidal *Salmonella* organism (confirmed by biochemical and serological means) was stored. The stored isolates were then sequenced, and the identity of these genomes were confirmed by multilocus sequence typing (MLST) and subsequently genotyped as described previously [7]. These were then linked to electronic patient records for relevant demographic and laboratory (complete blood counts, liver function tests and Widal serology) details after relevant ethics approvals [7].

### Results

**Fig. 1:**
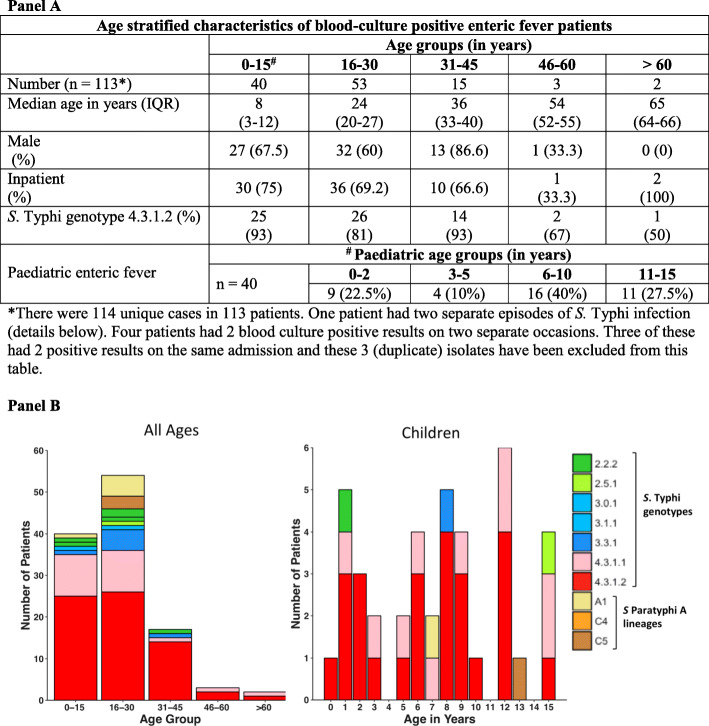
Age-stratified distribution of typhoidal *Salmonella* genotypes. Indian *S*. Typhi genotypes stratified according to age group. Individual *S.* Typhi genotypes and *S.* Paratyphi A lineages are coloured as described in the inset legend

Forty children (up to 15 years of age) and 73 adults had a culture-confirmed diagnosis of enteric fever during the study period. There were 114 unique isolates (100 *S.* Typhi and 14 *S*. Paratyphi A) from these 113 patients (one patient had two separate episodes of *S.* Typhi infection). The age distribution of patients (73 adults and 40 children) and infecting genotypes/serovars are illustrated in Fig. 1. There was no seasonal trend in disease occurrence indicating perennial transmission (Figure S1). Adults made up 65.5% of cases and 62.9% were male. The majority of cases occurred in children (age ≤ 15 years, *n* = 40, 35.1%) and young adults (age 16–30 years, *n* = 53, 46.5%; Fig. 1). The majority of patients were treated on an inpatient basis (68.9%), and there was no difference in serovar/genotype distribution among inpatients and outpatients nor in likelihood of admission to hospital between those infected with *S.* Typhi vs *S.* Paratyphi A (OR = 2.96, *p* = 0.15) or between children and adults (OR = 0.6, *p* = 0.25). The median duration of admission for adults was 4.5 days (IQR, 3–6), significantly longer than for children (2 days, IQR, 1–4; *p* = 0.002 using Wilcoxon’s rank sum test).

**Fig. 2:**
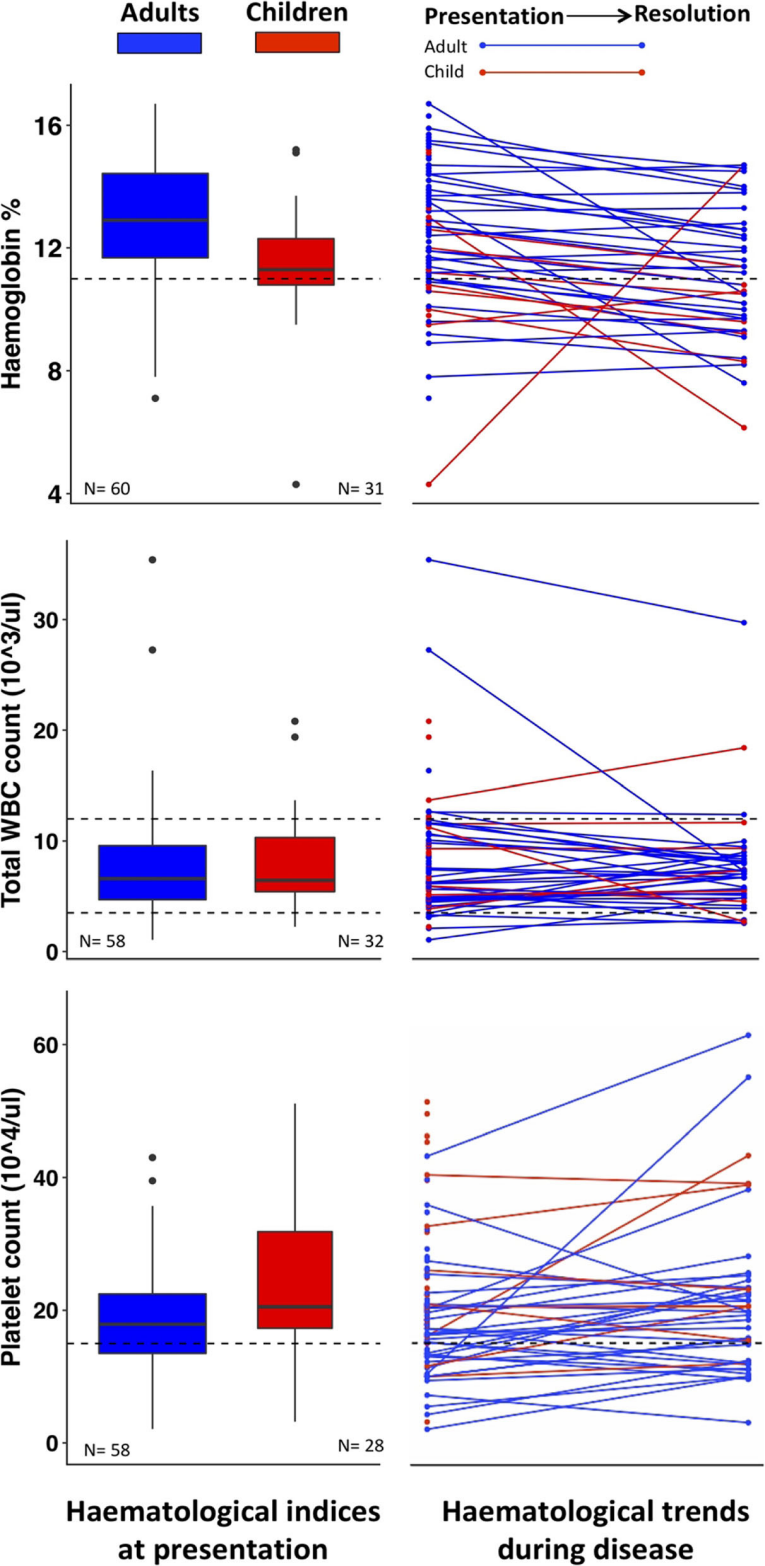
Haematological parameters of cases identified through this study. Haematological values of patients infected with typhoidal *Salmonella* on admission and trends over the course of disease according to inset legend. The dashed lines represent the cut-offs for the haematological parameters

Data on haemoglobin (Hb), total white blood cell (WBC) count and platelet count on presentation, stratified by adults and children, are illustrated in Fig. 2. Children (age ≤ 15 years) had significantly lower Hb values on presentation in comparison with adults (*p* = 0.002, Mann-Whitney *U* Test). A peripheral smear was done at the same time point as Hb estimation in 31 children and 24 adults, and only 3 children and 5 adults had a microcytic hypochromic (MCHC) blood picture. Leucopenia on admission was observed in 2 (6.1%) children and 8 (13.6%) adults while leucocytosis was seen in 8 (24.3%) and 9 (15.3%) children and adults, respectively. The WBC count did not differ significantly between adults and children on admission and, in the majority of cases, remained within the normal range until resolution of disease (Fig. 2). Two adults and two children had abnormally high WBC counts suggestive of a leukaemoid reaction. The two adults had repeated WBC counts during the course of the disease, and their leucocyte counts normalised with the initiation of cephalosporin treatment. Thrombocytopenia was seen in 24.7% of patients, and it was more common in adults but this difference was not significant.

Fifty-five patients had liver function tests at presentation. Raised liver enzymes were significantly more common among adult cases (*n* = 30, 54.5%) than paediatric cases (*n* = 5, 9%; *p* = 0.001 using Mann-Whitney *U* Test). The Widal test was undertaken at a single time point at presentation in all patients and was positive (above a titre of 1:160 for the O and H antigens) in only 68% of patients. Among children, 26 out of 37 infected with *S.* Typhi (70%) had a positive Widal test, vs 44 out of 66 (66%) adults (*p* = 0.6). There was also no association between Widal positivity and infecting genotype of *S*. Typhi. All patients made a full recovery with 14 days of third generation cephalosporin treatment which included intravenous ceftriaxone for 5–7 days and oral cefixime for the remaining days.

The genotypic population structure was dominated by the *S*. Typhi 4.3.1 genotypes, particularly the 4.3.1.2 lineage, with a number of prominent fluoroquinolone resistance molecular mechanisms as described previously [7]. Here, we describe the wider contextualisation of genetic relatedness of *S*. Typhi strains and the relation to clinical outcomes. A whole genome phylogeny for *S.* Typhi 4.3.1 (H58) was constructed comprising the 100 isolates from this study with 1133 from a global collection [5] (Figure S2). The strains isolated from countries of the Indian subcontinent clustered closely together, and the intermingling of isolates from adults and children suggests that transmission occurred between these groups as opposed to separate independent transmission cycles in adults and children. The strains clustered together irrespective of clinical outcomes such as anaemia, leucopoenia, leucocytosis, leukaemoid reaction, thrombocytopenia, abnormal liver enzymes and Widal positivity.

There were several noteworthy clinical scenarios observed over the course of the study period. A 26-year-old male patient presented with pain in the right hip. Clinical examination revealed decreased mobility at the hip joint along with painful movements, local rise in temperature and tenderness. A clinical diagnosis of septic arthritis was made (radiologic images illustrated in Figure S3) which was confirmed via bacteriologic culture of joint fluid aspirate that grew *S.* Typhi (genotype 4.3.1.2). This patient had no history of a haematologic or immunologic abnormality, and his peripheral smear at the time of diagnosis was normocytic and normochromic.

A 5-year-old child presented with fever and acute dyspnoea with clinical examination and preliminary laboratory findings suggestive of acute haemolysis. The child was found to have a haemoglobin (Hb) level of 4 g/dl in addition to a positive blood culture for *S.* Typhi (genotype 4.3.1.2). Further haematological workup of this patient revealed a diagnosis of hereditary spherocytosis based on a positive osmotic fragility test with a haemolytic episode precipitated by *S.* Typhi infection.

There were instances where *S*. Typhi was isolated from the same patient on two separate occasions. In 3 of these, the organism (*S*. Typhi genotype 4.3.1.2) was isolated during the same admission, > 48 h apart, despite initiation of injectable cephalosporin therapy, highlighting an increased clearance time despite the organism being susceptible to the antimicrobial. In all 3 of these cases, the isolates clustered tightly with other 4.3.1.2 isolates in the phylogenetic tree indicating no unique virulence features. In another instance, a confirmed diagnosis was made 2 months apart with two different *S.* Typhi genotypes, namely H58 lineage 2 (4.3.1.2) followed by a genotype 2.2.2 isolate 2 months later, possibly indicating an environmental risk for this individual.

### Discussion

These data provide insights into clinical spectrum of enteric fever in adults and children in an endemic region. The high-resolution analysis of pathogen genomic data along with clinical and laboratory data seems to suggest the modest role of pathogen virulence in determining clinical outcomes.

The large proportion of patients who were treated as inpatients is a possible reflection of disease severity, although this is also likely to be due to bias owing to the more rigorous testing in those patients who had clinical manifestations requiring inpatient care. It is interesting to note that adults had significantly longer duration of hospital admission when compared with children. This may indicate a more rapid clearance of bacteraemia in children, or it may be due to children presenting earlier with less severe disease due to concerned parents and subsequently having a lower threshold for admission. The perennial pattern of disease occurrence could suggest that the endemic nature of disease occurs through contamination of water supplies as opposed to point-source outbreaks.

The significant difference in Hb on admission between children and adults in this study was unexpected, and since the majority of these had a normocytic normochromic blood picture, it is likely that the anaemia was due to acute typhoid infection as opposed to nutritional deficiency. The relatively small proportion of patients with abnormal WBC counts was also surprising when compared with those of the human challenge model [8], where leucopoenia was observed shortly before the onset of clinical manifestations—in particular lymphopenia, neutropenia and eosinophilia [8]. This model used the same pathogen strain (genotype 3.1) to infect all participants, and the clinical as well laboratory features differed widely [8]. The difference in WBC count findings between volunteers in human challenge studies and this cohort of patients could be due a number of reasons including the discrepancy in sampling times and disease progression as well as repeated natural subclinical enteric fever infections leading to immune priming among patients in this study [9]. This may also be the reason for the non-reliability of the Widal test in endemic regions, particularly when it is done at a single time point as seen in this cohort of patients. Widal positivity rates were higher in children which may indicate a higher degree of immune naivety/lower rates of subclinical infections when compared with adults.

*Salmonella* bone and joint infections are more common with non-typhoidal *Salmonella* and most commonly seen in association with sickle cell disease [10]. The young adult in this study with septic arthritis was haematologically normal and had no prior right hip pathology. He made a full recovery with cephalosporin treatment, and follow-up after 6 months showed no residual joint pathology.

In the other case of a 5-year-old with hereditary spherocytosis, acute haemolysis in patients with structural red cell defects due to infection with *S.* Typhi has never been documented before, to our knowledge. Interestingly, the diagnosis of hereditary spherocytosis was made only after this infection. The patient received a blood transfusion during admission along with cephalosporin therapy and made a complete recovery. In both these cases, the organism cultured belonged to the 4.3.1.2 genotype and was fluoroquinolone non-susceptible with two QRDR SNPs (*gyrA* and *par*) in the first case and three QRDR SNPs in the second case. Both these isolates also clustered tightly within the local *S.* Typhi 4.3.1.2 population (Figure S2) suggesting they are typical of locally circulating strains.

Although mathematical models suggest about 5 childhood infections are required to induce life-long protection [11], this has not been demonstrated in the field or in experimental laboratory settings and has important implications when designing vaccination schedules in terms of interval between doses and selection of vaccine antigens. A 28-year-old male among the cohort of cases in this study was treated on an outpatient basis in April 2017. The infecting agent was a 4.3.1.2 genotype strain. Two months later in June 2017, he was again diagnosed with enteric fever and the infecting genotype was 2.2.2. On both occasions, the patient was treated with a cephalosporin and made a complete recovery. It is not known whether the difference in genotype is sufficient to escape natural immunity and allow a second infectious episode, or whether this would have occurred regardless of the infecting genotype.

Our study has limitations as all isolates examined were from a single hospital-based passive surveillance programme and thus may not be representative of the disease trends in the wider community. The laboratory data analysis included only routine haematological and laboratory tests at admission and discharge. We also did not actively survey all febrile cases coming to the hospital, and it is known that hospital-based active surveillance of enteric fever yields a higher number of cases [12]. However, the age distribution trends observed here are similar to those seen in larger community-based studies from the region [3] and support the idea that vaccination strategies should be aimed at children and young adults in endemic regions.

## Conclusions

These data constitute the first study of blood culture-confirmed enteric fever in India linking clinical features and high-resolution pathogen genomics. In Southern India, enteric fever continues to be a public health issue with non-specific and sometimes atypical presentations, likely driven by the host-response and environmental factors. Children and young adults account for a substantial proportion of the disease burden with certain clinical features differing between children and adults.

## Supplementary information

**Additional file 1: Figure S1**. Temporal distribution of typhoidal *Salmonella* serovars. Representation of typhoidal serovars, treatment status and genotype distribution as per the inset legends between adults and children over the study period. **Figure S2**. Phylogenetic tree of 4.3.1 (H58) *S.* Typhi isolates from Bengaluru and a global collection. This tree is made up of all 100 4.3.1 isolates from this study plus 1133 globally representative 4.3.1 isolates, and the rings are coloured according to the inset legend. The intermingling of isolates of children and adults is evident from the coloured branches and ring1. Branch lengths are indicative of the estimated number of substitutions rate per variable site; the tree was outgroup rooted using *S.* Paratyphi A strain AKU_12601. **Figure S3**. Radiological imaging of the right hip joint of a patient with septic arthritis due to *S*. Typhi (red arrows point to regions of interest as mentioned in the description of each panel). Panel A: CT image, transverse section, showing irregularity and erosions due to the infective process with no obvious breach in cortex. Panel B: MRI image, transverse section, showing involvement of the right iliac crest ,acetabular roof, head of the femur (cystic change), greater and lesser trochanter. Panel C: MRI image, T2W coronal view, shows the extent of bony involvement with effusion in the joint space. Panel D: MRI image, T2W, shows inflammation of the surrounding soft tissue including right iliacus muscle. Left hip joint appears normal.

## Data Availability

All data generated or analysed during this study are included in this published article [and its supplementary information files] as well as in the European Nucleotide Archive under project PRJEB14050.

## Abbreviations

*S.* Typhi: *Salmonella enterica* serovar Typhi
*S.* Paratyphi A: *Salmonella enterica* serovar Paratyphi A
MLST: Multilocus sequence typing
Hb: Haemoglobin
WBC: Total white blood cell
MCHC: Microcytic hypochromic
