# Supplementary material for: Pathogen genomic surveillance of typhoidal Salmonella infection in adults and children reveals no association between clinical outcomes and infecting genotypes
Source: Trop Med Health. 2020 Jul 13;48:58. doi: 10.1186/s41182-020-00247-2 (PMC7359007; doi:10.1186/s41182-020-00247-2)

**Supplementary Figure 1**. **Temporal distribution of typhoidal *Salmonella***

Representation of typhoidal serovars, treatment status and genotype distribution as per the inset legends between adults and children over the study period. The bottom panel of the figure has been adapted from *Britto et al,* 2020.


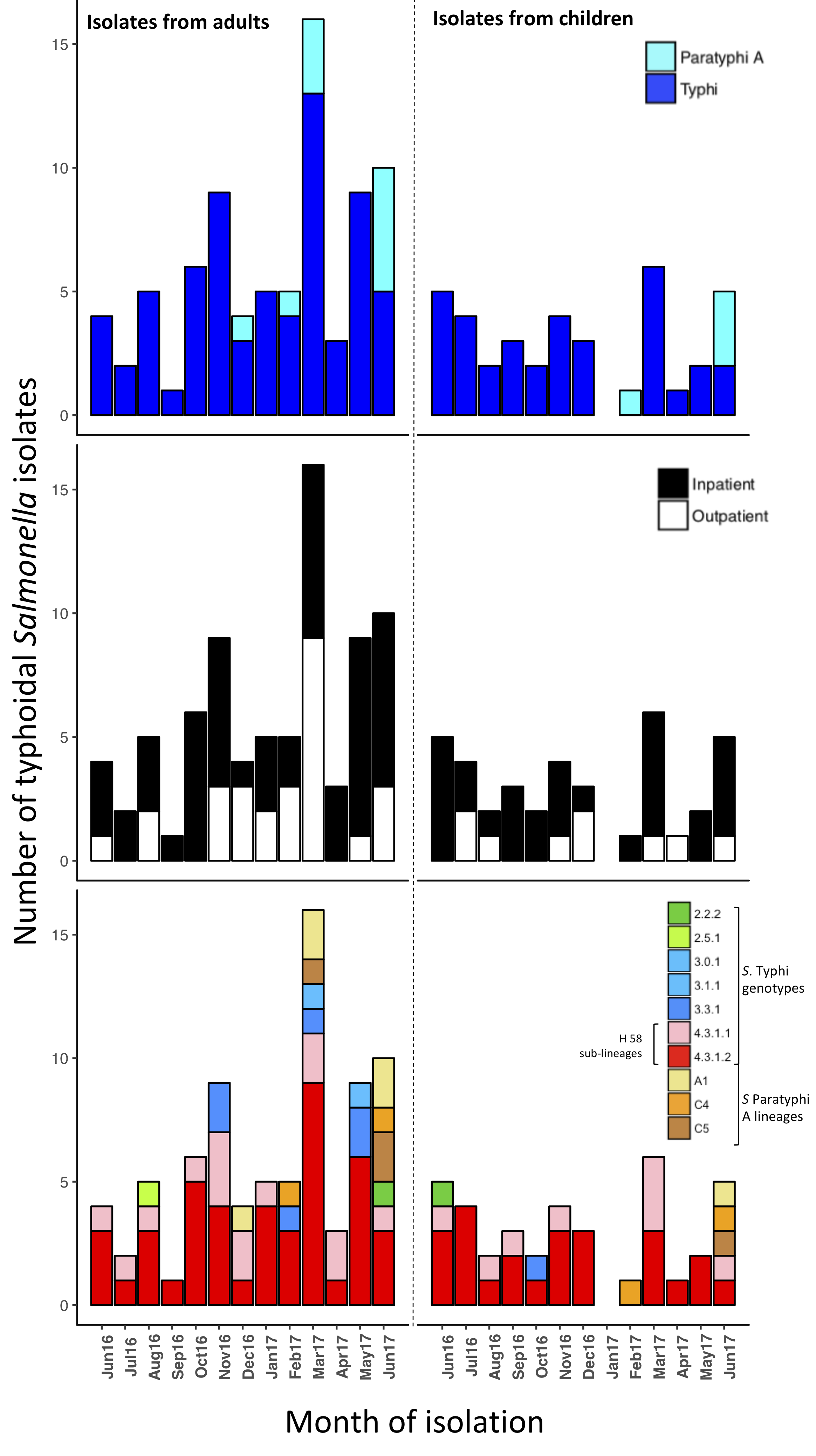


**Supplementary Figure 2. Phylogenetic tree of 4.3.1 (H58) *S.* Typhi isolates from Bengaluru and a global collection**

This tree is made up of all 100 4.3.1 isolates from this study plus 1133 globally representative 4.3.1 isolates, and the rings are coloured according to the inset legend. The intermingling of isolates of children and adults is evident from the coloured branches and ring1. Branch lengths are indicative of the estimated number of substitutions rate per variable site; the tree was outgroup rooted using *S.* Paratyphi A strain AKU_12601.


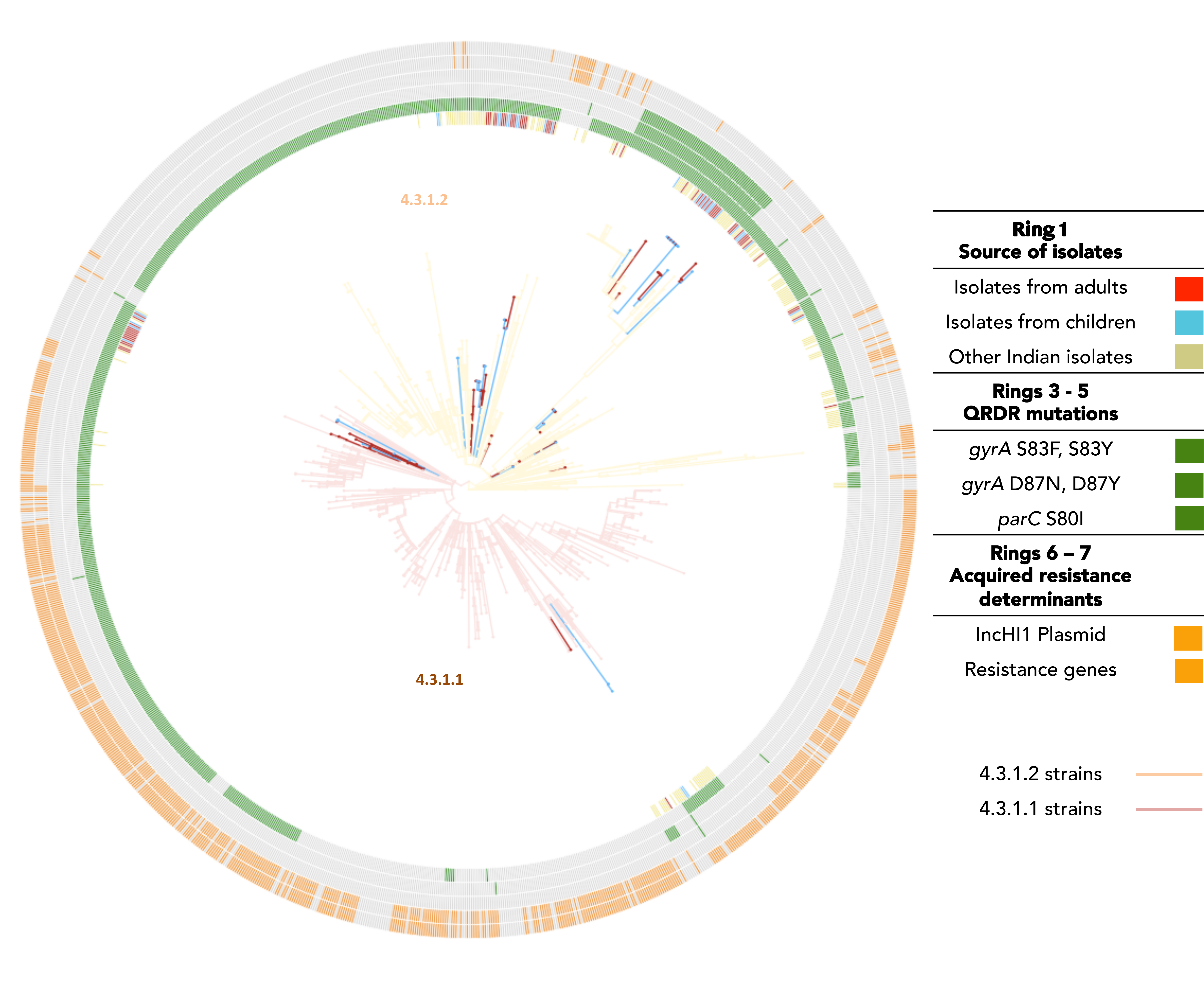


**Panel A**: CT image, transverse section, showing irregularity and erosions due to the infective process with no obvious breach in cortex.

**Panel B**: MRI image, transverse section, showing involvement of the right iliac crest ,acetabular roof, head of the femur (cystic change), greater and lesser trochanter

**Panel C**: MRI image, T2W coronal view, shows the extent of bony involvement with effusion in the joint space.

**Panel D**: MRI image, T2W, shows inflammation of the surrounding soft tissue including right iliacus muscle

Left hip joint appears normal.

**Supplementary Figure 3.** **Radiological imaging of the right hip joint of a patient with septic arthritis due to *S*. Typhi** (red arrows point to regions of interest as mentioned in the description of each panel)


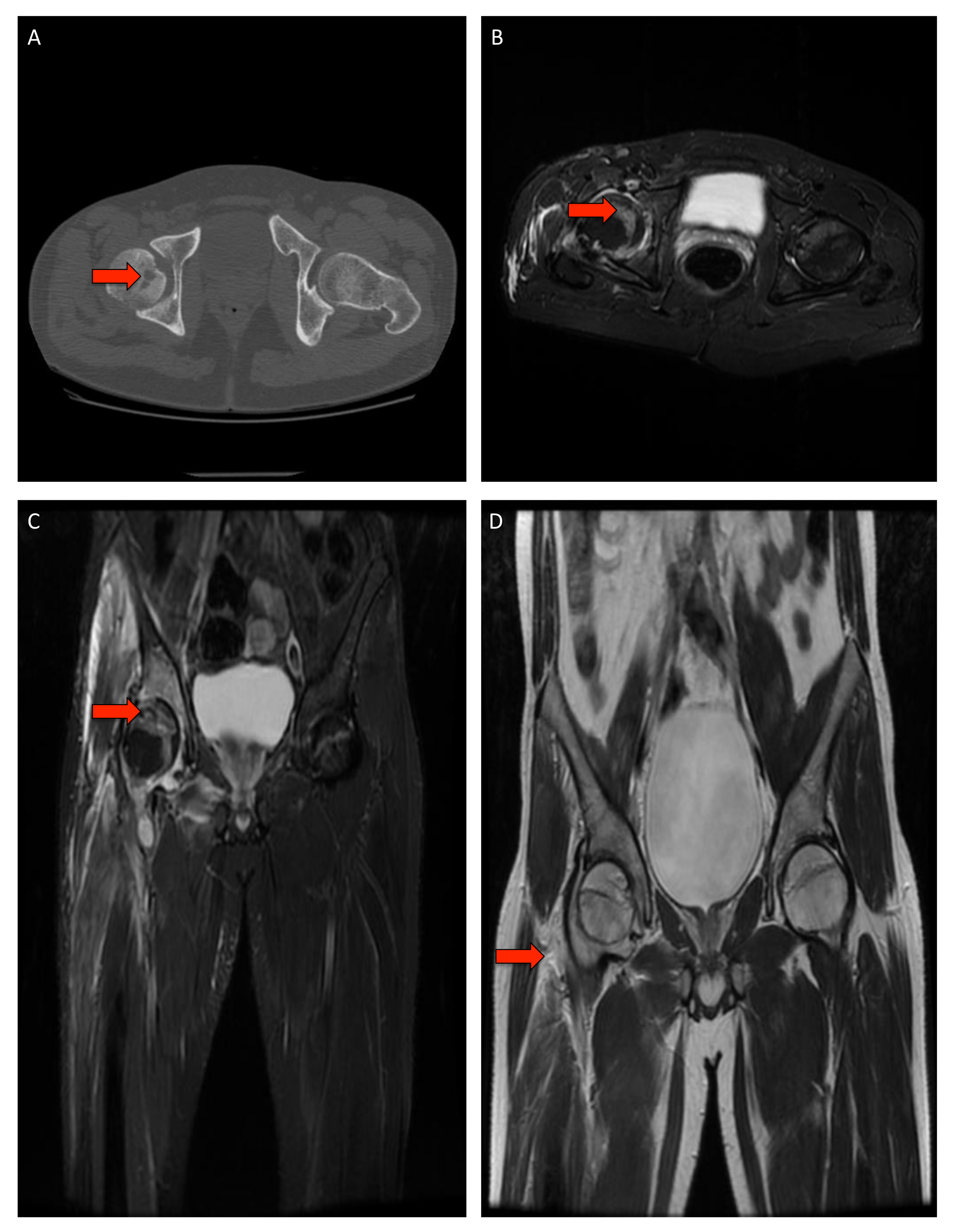

Supplement: Supplementary file 1 — Additional file 1: Figure S1. Temporal distribution of typhoidal Salmonella serovars. Representation of typhoidal serovars, treatment status and genotype distribution as per the inset legends between adults and children over the study period. Figure S2. Phylogenetic tree of 4.3.1 (H58) S. Typhi isolates from Bengaluru and a global collection. This tree is made up of all 100 4.3.1 isolates from this study plus 1133 globally representative 4.3.1 isolates, and the rings are coloured according to the inset legend. The intermingling of isolates of children and adults is evident from the coloured branches and ring1. Branch lengths are indicative of the estimated number of substitutions rate per variable site; the tree was outgroup rooted using S. Paratyphi A strain AKU_12601. Figure S3. Radiological imaging of the right hip joint of a patient with septic arthritis due to S. Typhi (red arrows point to regions of interest as mentioned in the description of each panel). Panel A: CT image, transverse section, showing irregularity and erosions due to the infective process with no obvious breach in cortex. Panel B: MRI image, transverse section, showing involvement of the right iliac crest ,acetabular roof, head of the femur (cystic change), greater and lesser trochanter. Panel C: MRI image, T2W coronal view, shows the extent of bony involvement with effusion in the joint space. Panel D: MRI image, T2W, shows inflammation of the surrounding soft tissue including right iliacus muscle. Left hip joint appears normal. [file 41182_2020_247_MOESM1_ESM.docx]
